# Effect of Combined Dasatinib and Fisetin Treatment on Senescent Cell Clearance in Monkeys

**DOI:** 10.1093/geroni/igaa057.432

**Published:** 2020-12-16

**Authors:** Ricki Colman, Tamara Tchkonia, Tamar Pirtskhalava, Nino Giorgadze, Larissa Prata, Kalli Schaefer, James Kirkland

**Affiliations:** 1 University of Wisconsin, Madison, Madison, Wisconsin, United States; 2 Mayo Clinic, Rochester, Minnesota, United States

## Abstract

Aging is the biggest risk factor for the most serious chronic diseases and disabilities. Cellular senescence, a state in which cells stop dividing but release factors that damage other cells, may contribute to both age-related and chronic diseases. Removal of senescent cells from aged mice has been shown to delay aging and age–related disabilities. Our goal was to determine the ability of potential senolytic agents to remove senescent cells in a primate model. Several agents and combinations were tested including Fisetin, Navitoclax, combined Dasatinib and Quercetin, and combined Dasatinib and Fisetin. Here we describe the Dasatinib and Fisetin trial. Dasatinib is an FDA approved oral anticancer drug that has been used to treat chronic myelogenous leukemia in humans. Fisetin is a flavonoid that can be found in many plants, particularly strawberries, and acts as a coloring agent. After baseline measurements, six older (mean age=21 years) female rhesus monkeys (Macaca mulatta) were given a combined oral dose of Dasatinib (5 mg/kg) and Fisetin (100 mg/kg) on two consecutive days. Animals were additionally assessed at 1- and 7-weeks following dosing. At 7 weeks post dosing, there were fewer (p<0.05) p16+ cells in the epidermis compared to baseline. Similarly, there was a reduction (p<0.05) in p21+ cells in the epidermis at 1- and 7-weeks post dosing compared to baseline. There were no negative outcomes associated with treatment. This study provides preliminary evidence for the senolytic potential of combined Dasatinib and Fisetin treatment and indicates that pharmacological mitigation of age-related changes is possible.

